# Acantholytic Dyskeratosis Consistent With Grover's Disease After Letrozole Therapy

**DOI:** 10.7759/cureus.54262

**Published:** 2024-02-15

**Authors:** Osman Mahboob, Yusuf Amawi, Mohammad Tahseen Alkaelani, Omar Mahboob, Cynthia Tie

**Affiliations:** 1 Clinical Sciences, Florida State University College of Medicine, Tallahassee, USA; 2 Dermatology, Family Dermatology of North Florida, Tallahassee, USA

**Keywords:** autoimmune flare up, chemotherapeutics, grovers disease, letrozole, acantholytic dyskeratosis

## Abstract

We present a rare case of Grover's disease (GD) associated with letrozole therapy in a 66-year-old female with stage IV breast cancer. GD is a dermatological condition characterized by papulovesicular lesions typically found on the chest and trunk. While GD is linked to chemotherapeutic agents, its association with letrozole is not well documented. The patient presented with a pruritic rash on her neck, right arm, and trunk, initially misdiagnosed as contact dermatitis. Despite treatment with triamcinolone acetonide, the rash persisted. A subsequent punch biopsy confirmed acantholytic dyskeratosis consistent with GD. Discontinuation of letrozole and treatment with augmented betamethasone dipropionate resulted in significant improvement within four weeks. This case underscores the importance of considering drug-induced dermatological conditions in patients undergoing chemotherapy. While hypersensitivity drug eruptions typically present as morbilliform-patterned cutaneous eruptions, GD should be considered, especially in older patients with multiple medications. The incidence of GD following letrozole therapy is not well studied, making this case a valuable addition to the limited literature. In summary, recognizing and managing drug-induced skin conditions in cancer patients are crucial. This report contributes to our understanding of the potential association between letrozole and GD, emphasizing the need for further research in this area.

## Introduction

Grover’s disease (GD) is an acquired dermatosis characterized by fragile vesicles that progress into erythematous, crusted, and keratotic erosions. Histologically, GD is characterized by focal acantholysis, frequently combined with dyskeratosis [1]. The distribution primarily involves the trunk and proximal extremities [2]. While many patients remain asymptomatic, others experience severe pruritus. The etiology of GD remains poorly understood, and although commonly observed as an incidental finding in healthy individuals, it has rarely been associated with certain chemotherapeutic agents such as daunorubicin, etoposide, and docetaxel [3]. In this report, we present the case of a 66-year-old female who presented to a dermatology clinic with GD subsequent to treatment with the chemotherapeutic letrozole.

## Case presentation

**Figure 1:**
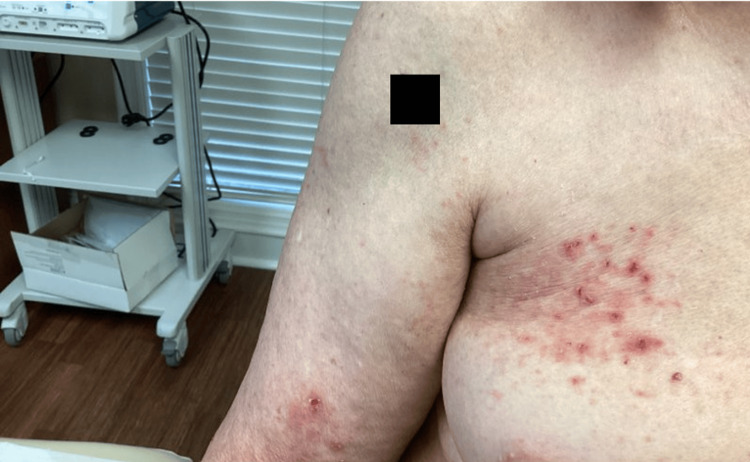
Gross image of the patient's right arm and breast revealing the eczematous patches and erythematous papules consistent with a presentation of GD. GD: Grover's disease.

A 66-year-old female with a history of stage IV breast cancer, who is on letrozole, presented with a three-week history of a rash on her neck, right arm, and trunk. The rash was mildly pruritic. The patient denied starting any new medication other than letrozole 2.5 mg for her stage IV breast cancer. A physical exam revealed geometric eczematous patches and excoriated pink papules distributed on the neck, right arm, and trunk (Figure 1).

An initial diagnosis of contact dermatitis was made, and the patient was prescribed triamcinolone acetonide 0.1% ointment to be applied topically to the affected areas twice daily and was told to follow up in four weeks.

**Figure 2:**
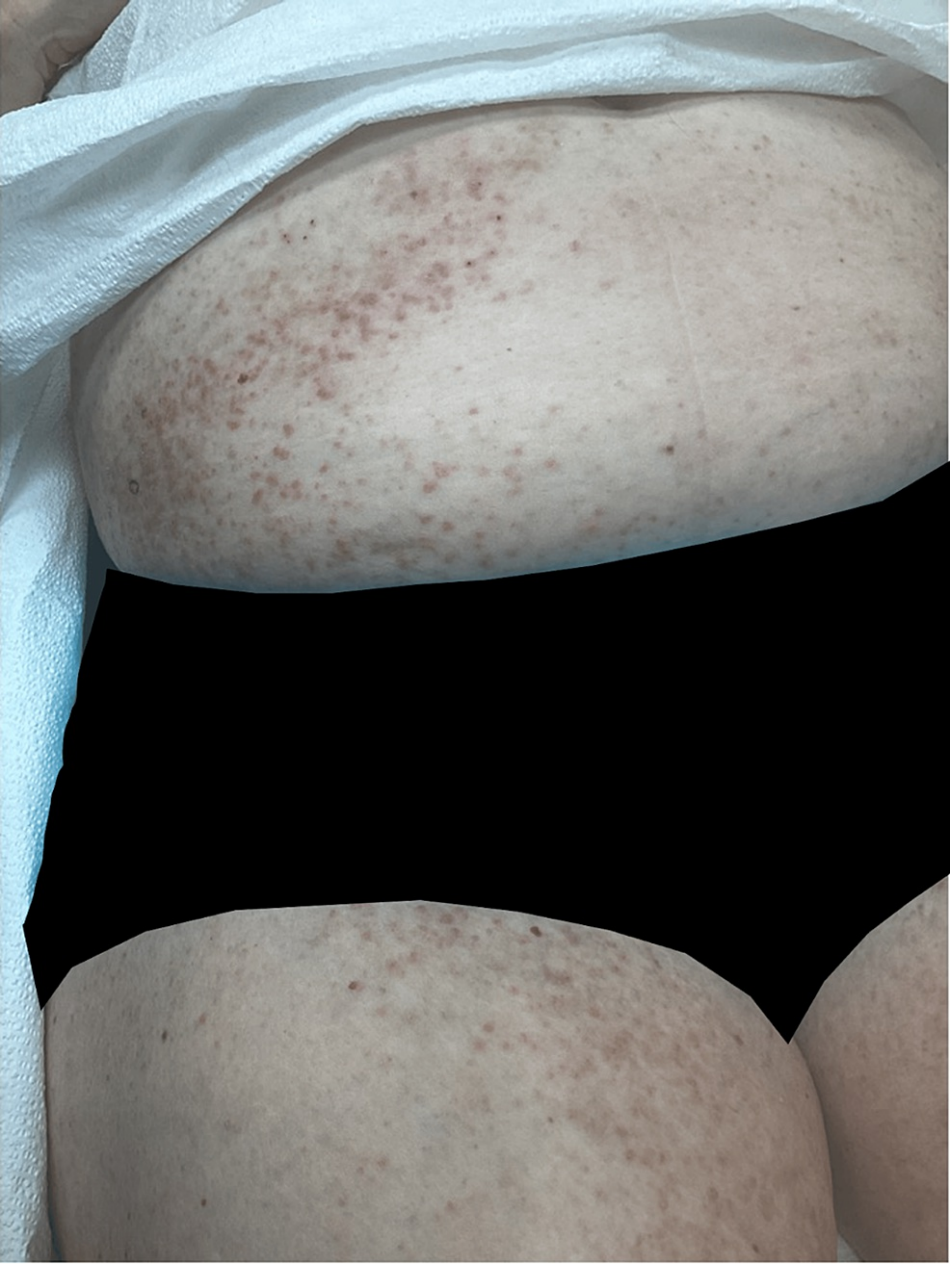
Image of the patient's abdomen and thighs showing erythematous papules diffusely spread consistent with a presentation of GD. GD: Grover's disease.

Upon return to the office, the patient's rash did not improve. A physical exam revealed multiple papules, with some being follicular and others more reticulated on the abdomen, inner thighs, and inframammary region (Figure 2).

**Figure 3:**
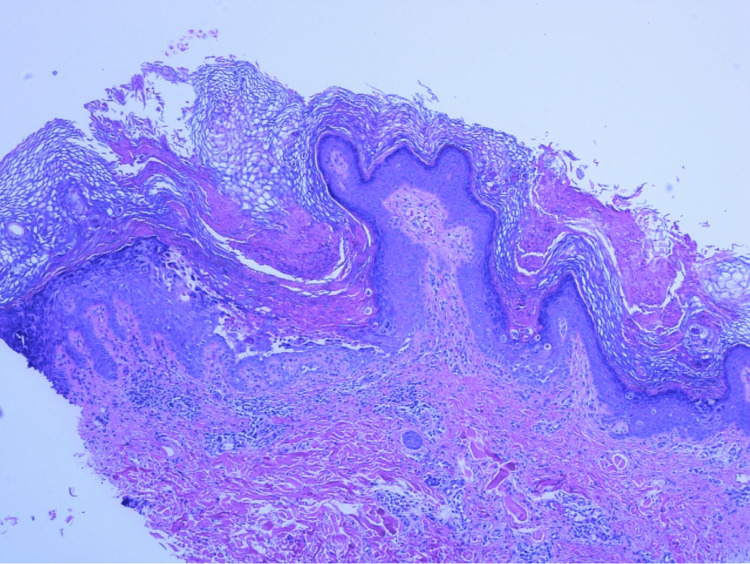
Biopsy of the patient's right lower abdomen stained with hematoxylin and eosin showing acantholytic dyskeratosis with suprabasilar acantholysis a pathological hallmark of GD. GD: Grover's disease.

A punch biopsy of a lesion on the right lower abdomen was performed and showed acantholytic dyskeratosis with suprabasilar acantholysis present. The lesions and clinical picture were consistent with a diagnosis of GD (Figure 3).

The patient was given augmented betamethasone dipropionate 0.05% ointment to be applied topically to affected areas twice daily and was told to follow up. The patient reported that the rash improved with the betamethasone ointment within four weeks of use and that upon cessation of letrozole, her rash completely cleared up.

## Discussion

Targeted chemotherapeutic drugs of various classes have been implicated in a wide range of adverse mucocutaneous lesions. The majority of instances of hypersensitivity drug eruptions manifest as morbilliform-patterned cutaneous eruptions, constituting approximately 95% of all skin rashes linked to drug use [4]. Adverse reactions to systemic medications frequently result in eczematous responses. More acutely, geriatric patients are particularly susceptible to such reactions, likely as a result of concurrent medication use, changes in drug metabolism, and progressive degeneration of the skin [5]. It is important to note that drug eruptions are also known to mimic the presentation of a wide range of dermatological conditions and as such may be misidentified [6].

As previously stated, GD is a rare dermatological condition (incidence: <1%) markedly known to be an acquired, idiopathic eruption of papulovesicular lesions typically presenting on the chest and trunk [7]. Among the studied cases, GD is more commonly seen in males (2.4:1) and Caucasian patients (74%), with an average onset at 61 years of age [8]. Although little is known about the pathoetiology of GD, its relationship with malignancy and chemotherapy has been highlighted in a 2017 study by Gantz et al. that reviewed 69 cases of GD finding that 61% of cases experienced previous malignancy and of that specific subset, 62% had been treated with some form of chemotherapy [9]. Although primarily indicated by pathological findings, the studied histopathological examinations of the skin biopsy further indicate a clear diagnosis of GD in our presented case.

While chemotherapeutic drugs are often implicated in drug eruptions and GD, the incidence of the aromatase inhibitor letrozole resulting in GD has not been widely studied. To uncover similar clinical presentations, a comprehensive literature review was performed. The study conducted by Tripathy et al. shed light on a singular case that exhibited a striking resemblance to the current case under examination [10]. However, in the former case, a comprehensive biopsy was not conducted, thereby introducing an element of conjecture regarding the precise pathological character of the drug-induced eruption. Tripathy et al. asserted their pioneering contribution in delineating a drug eruption provoked by letrozole, thereby highlighting the infrequency of such clinical manifestations within the medical domain [10]. Cases of this nature serve to indicate the importance of making effective clinical connections in hopes of alleviating symptoms and improving patient quality of life while they receive chemotherapeutic treatments.

## Conclusions

Physicians should be aware of the possibility of inducing drug eruptions such as GD when placing patients on medication therapy. The development of GD after letrozole has only been discussed once previously in the literature, as mentioned in the discussion. This case highlights the need to consider drug eruptions, such as GD, when a patient presents for dermatologic evaluation and the need to consider alternative therapeutic options when such eruptions occur.
